# The Association of Resting Heart Rate with the Presence of Diabetes in Korean Adults: The 2010-2013 Korea National Health and Nutrition Examination Survey

**DOI:** 10.1371/journal.pone.0168527

**Published:** 2016-12-16

**Authors:** Jae Won Hong, Jung Hyun Noh, Dong-Jun Kim

**Affiliations:** Department of Internal Medicine, Ilsan-Paik Hospital, College of Medicine, Inje University, Koyang, Gyeonggi-do, Republic of Korea; Nagoya University, JAPAN

## Abstract

**Background:**

Previous epidemiologic studies have shown that elevated resting heart rate (HR) is associated with higher cardiovascular disease (CVD) morbidity and mortality. Although the relationship between elevated HR and CVD is well established, the association between resting HR and diabetes has been relatively understudied, particularly in non-Western populations.

**Objectives:**

We confirmed the association between the presence of type 2 diabetes and resting HR in the Korean adult population using data from the 2010–2013 Korea National Health and Nutrition Examination Survey (KNHANES).

**Methods:**

Among 25,712 adults (≥ 19 years of age) who participated in the 2010–2013 KNHANES, a total of 22,512 subjects completed laboratory examinations and were included in this analysis. The fasting plasma glucose (FPG) level was categorized into the following five groups: normal fasting glucose (NFG) 1 (<90 mg/dL), NFG 2 (90–99 mg/dL), impaired fasting glucose (IFG) 1 (100–110 mg/dL), IFG 2 (111–125 mg/dL), and diabetes (≥ 126 mg/dL).

**Results:**

The unadjusted weighted resting HRs were 69.6, 69.4, 69.8, 70.1, and 72.0 beats per minute (bpm) in the NFG 1, NFG 2, IFG 1, IFG 2, and diabetes groups, respectively (P<0.001). We assessed the adjusted weighted resting HR according to the FPG level after adjusting for age, sex, smoking history, high risk alcohol drinking, daily energy intake, waist circumference, serum total cholesterol level, serum triglyceride (TG) level, serum white blood cell (WBC) count, serum hemoglobin (Hb), and the presence of hypertension. The adjusted weighted resting HR significantly increased across the FPG groups (P<0.001). The weighted prevalence rates of diabetes were 6.8% (6.2–7.5%), 7.6% (6.7–8.5%), 8.0% (7.0–9.1%), and 11.8% (10.8–12.7%) in subjects with HR ≤ 64, 65–69, 70–75, and ≥ 76 bpm, respectively (P<0.001), after adjusting for the confounding factors mentioned above. Using resting HR ≤ 64 bpm as the control, resting HR ≥ 76 bpm was correlated with the presence of diabetes (adjusted OR 1.83, 95% CI 1.55–2.16, P<0.001). Each 10 bpm increment of HR increased the risk of the presence of diabetes by 35% (P<0.001). This association of high resting HR with the presence of diabetes was not influenced by the status of blood pressure (BP) medication.

**Conclusion:**

We demonstrated that higher HR was associated with diabetes in a representative sample of Korean adults. These positive associations were independent of age, sex, current smoking, high risk alcohol drinking, daily energy intake, waist circumference, and the presence of hypertension and other potential confounders. This study suggests that individuals with higher resting HR are at risk of diabetes and that HR might provide an easy and simple surrogate marker for the risk of diabetes.

## Introduction

Heart rate (HR) is an important vital sign, which can be easily measured without special equipment. HR reflects myocardial oxygen, coronary blood flow, and adaptation of cardiac output to metabolic needs [1]. It is regulated by complex interactions of multiple mechanisms, including sympathetic and parasympathetic nervous system, the neurohormonal system, and the environment [2].

Previous epidemiologic studies have shown that elevated resting HR is associated with higher cardiovascular disease (CVD) morbidity and mortality [3–5]. This association may be explained by sympathetic over-activity, which has been also linked with hypertension, obesity, and metabolic syndrome, all of which increases the risk of type 2 diabetes [6–8].

In the context of this background, emerging evidence suggests that higher HR may also be associated with increased risk of type 2 diabetes [9–13]. A recent meta-analysis, including 10 cohort studies, showed that a high vs. low resting HR was associated with an 83% increase in the relative risk of type 2 diabetes [9]. However, some studies did not found the significant association between type 2 diabetes and increased heart rate after adjustment for potential confounding factors [14–16].

Although the relationship between elevated HR and CVD is well established, the association between resting HR and diabetes has been relatively understudied, particularly in non-Western populations. Recently, Yang *et al*. showed that resting HR had a significant positive dose response association with diabetes and metabolic syndrome, using 2010–2012 Korea National Health and Nutrition Examination Survey (KNHANES) [17]. However, there has been little research on the relationship between resting HR and fasting plasma glucose (FPG) level, as well as the presence of diabetes, using nationally representative data.

Herein, we assessed the resting HR according to the FPG level and confirmed the association between the presence of type 2 diabetes and resting HR in the Korean adult population using the data from the 2010–2013 KNHANES.

## Methods

### Study population and data collection

This study is based on data from the 2012–2013 KNHANES, a cross-sectional and nationally representative survey conducted by the Korea Centers for Disease Control and Prevention (KCDC). As described in detail previously [18], KNHANES is composed of independent dataset from the general population of Korea, similar to the National Health and Nutrition Examination Survey in the United States (NHANES).

Daily energy intake was obtained from the nutrition survey, as a part of KNHANES [19]. A single twenty-four hour dietary recall was collected from each respondent through in-person interviewers. Recipes for all food items consumed were also collected. To enhance recall, particularly for away-from-home food consumption, actual food shapes and two-dimensional models of plates and bowels were used to assist the respondent’s report of the volumes of the food items consumed. Based on the recipes collected from each household during the interview, the weight of each ingredient was estimated from the volume of food ingested [20].

Alcohol consumption was assessed by drinking behavior during the month before the interview. To assess heavy alcohol consumption more accurately, we introduce the Alcohol Use Disorders Identification Test (AUDIT), which was developed by the World Health Organization (WHO) as a simple method of screening for excessive drinking [21]. The AUDIT comprises three domains: hazardous alcohol use (frequency of drinking, typical quantity, and frequency of heavy drinking), dependence symptoms (impaired control over drinking, increased salience of drinking, and morning drinking), and harmful alcohol use (guilt after drinking, blackouts, alcohol-related injuries, and other concerns about drinking). In most cases, the total AUDIT score will reflect the patient’s level of risk related to alcohol.

AUDIT scores were categorized into three groups according to the WHO guidelines: low-risk, 0 to 7 points; intermediate-risk, 8 to 15 points; and high-risk, ≥16 points. AUDIT scores of ≥16 represented high-level alcohol problems, suggesting the need for counseling and continued monitoring or further diagnostic evaluation for alcohol dependence.

Hypertension was defined as systolic blood pressure (BP) ≥ 140 mmHg, diastolic BP ≥ 90 mmHg, or use of antihypertensive medication irrespective of BP. Diabetes was defined as a FPG level ≥ 7.0 mmol/L, current anti-diabetes medication usage, or a previous diagnosis of diabetes by a physician.

HR per minute was determined by counting the number of beats on the subject’s wrist for 15 seconds and multiplying this number by 4. Taking into account the distribution of HR, we classified our participants into four groups (HR ≤ 64 [n = 8,762], 65–69 [n = 3,926], 70–75 [n = 3,659], and ≥ 76 [n = 6,165] beats per minute (bpm)).

### Laboratory methods

Blood samples were collected in the morning after fasting for at least 8 h. Total cholesterol, FPG, triglyceride (TG), and high-density lipoprotein (HDL)-cholesterol levels were measured using a Hitachi Automatic Analyzer 7600 (Hitachi, Tokyo, Japan). HbA1c was measured using high-performance liquid chromatography (HLC-723G7; Tosoh, Tokyo, Japan). The hemoglobin (Hb) level was measured via the cyanide-free sodium lauryl sulfate Hb detection method, using an XE-2100D hematology analyzer (Sysmex, Kobe, Japan).

### Ethics statement

This study was approved by the Institutional Review Board (IRB 2016-04-009) of Ilsan Paik Hospital, Republic of Korea. After approval of the study proposal, the KNHANES dataset was made available at the request of the investigator. Because the dataset did not include any personal information, and participants’ consent had already been given for KNHANES, our study was exempt from the requirement for participant consent.

### Statistical analyses

The KNHANES participants were not sampled randomly. The survey was designed using a complex, stratified, multistage probability-sampling model; thus, individual participants were not equally representative of the Korean population. To obtain representative prevalence rates from the dataset, it was necessary to consider the power of each participant (sample weight) as representative of the Korean population. Following approval from the KCDC, we received a survey dataset that included information regarding the survey location; stratification by age, sex, and various other factors; and the sample weight for each participant. The survey sample weights, which were calculated by taking into account the sampling rate, response rate, and age/sex proportions of the reference population (2005 Korean National Census Registry), were used in all of the analyses to provide representative estimates of the non-institutionalized Korean civilian population.

Difference in age according to resting HR was evaluated by analysis of variance, and the percentage of females was evaluated by the χ^2^-test. To compare the age- and sex-adjusted weighted clinical characteristics according to resting HR, analysis of covariance (ANCOVA) and the Bonferroni *post hoc* test were used. General linear models were used to assess the weighted and adjusted prevalence of diabetes according to resting HR after adjustment for confounders (Models 1–3). Age, sex, smoking, high risk alcohol drinking (AUDIT scores of ≥16), and daily energy intake were adjusted in Model 1. In Model 2, waist circumference, serum TG, serum total cholesterol, serum white blood cell (WBC) count, serum Hb, and the presence of hypertension were adjusted for, in addition to the parameters in Model 1. Adjustment for confounding factors in Model 3 was performed after exclusion of subjects taking anti-hypertensive medication. General linear models were used to assess weighted resting HR according to FPG levels before (Model 1) and after (Models 2–5) adjustment for confounders. Age and sex were adjusted for in Model 2. In Model 3, current smoking, high risk alcohol drinking, and daily energy intake, as well as age and sex, were adjusted for in the analysis. In Model 4, waist circumference, serum TG, serum total cholesterol, serum WBC, serum Hb, and the presence of hypertension were adjusted for, in addition to the parameters in Model 3. Adjustment for confounding factors in Model 4 was performed after the exclusion of subjects taking anti-hypertensive medication in Model 5.

We classified FPG levels into the following five groups: normal fasting glucose (NFG) 1 (< 90 mg/dL), NFG 2 (90–99 mg/dL), impaired fasting glucose (IFG) 1 (100–110 mg/dL), IFG 2 (111–125 mg/dL), and diabetes, defined as either FPG ≥ 126 mg/dL, treatment with anti-diabetes drugs, or a history of diabetes diagnosed by a physician.

Logistic regression analysis was used to evaluate the odds ratios (ORs) of resting HR for the presence of diabetes according to age, sex, and the presence of BP medication, using the variables mentioned above as covariates. All of the tests were two-sided, and P values < 0.05 indicate statistical significance. Statistical analyses were performed using SPSS for Windows software (ver. 21.0; SPSS Inc., Chicago, IL, USA).

## Results

### Weighted demographics and clinical characteristics of the study population

The weighted demographics and clinical characteristics of the study population are presented in Table 1. Among 25,712 adults (≥ 19 years of age) who participated in the 2010–2013 KNHANES, a total of 22,512 subjects completed laboratory examinations and were included in this analysis.

**Table 1:** Demographic and clinical characteristics of Korean population, aged 19 years and older, in 2010–2013 Korea National Health and Nutrition Examination Survey.

	unweighted	weighted
N	22,512	36,466,065
Age (years)	50 (19–97)	45(19–97)
Women (%)	56.8	49.8 ± 0.3
Current smoking (%)	20.1	25.4 ± 0.4
Alcohol drinking ≥ x4/week (%)	6.6	6.9 ± 0.2
AUDIT score	6.2 ± 0.1	7.0 ± 0.1
Alcohol drinking risk (low/intermediaate/high risk)%	73.2/17.2/9.6	67.7 ± 0.4/ 20.6 ± 0.3/ 11.7 ± 0.3
Exercise ≥ x 3/week (%)	18.2	18.8 ± 0.4
College graduation (%)	30.1	32.1 ± 0.6
Daily energy intake (kcal/day)	1987 ± 6	2070 ± 10
Body mass index (kg/m^2^)	23.7 ± 0.1	23.7 ± 0.1
Waist circumference (cm)	81.0 ± 0.1	80.9 ± 0.1
Systolic BP (mmHg)	119.3 ± 0.1	117.7 ± 0.2
Diastolic BP (mmHg)	76.0 ± 0.1	76.2 ± 0.1
Anti-hypertensive medication (%)	19.3	14.0 ± 0.3
Hypertension (%)	31.0	25.9 ± 0.4
Resting heart rate (bpm)	69.6 ± 0.1	69.8 ± 0.1
Fasting plasma glucose (mg/dl)	98.2 ± 0.1	97.2 ± 0.2
Anti-diabetes medication (%)	6.9	5.3 ± 0.2
Impaired fasting glucose (%)	20.0	18.8 ± 0.4
Diabetes (%)	10.1	8.4 ± 0.2
Serum total cholesterol (mg/dl)	189.3 ± 0.2	187.8 ± 0.4
Serum triglyceride (mg/dl)	133.0 ± 0.7	134.8 ± 1.1
Serum HDL-cholesterol (mg/dl)	52.5 ± 0.1	52.5 ± 0.1
Serum White blood cell (/mm3)	6042 ± 114	6123 ± 169
Serum hemoglobin (g/dl)	14.0 ± 0.1	14.2 ± 0.1
Self-reported CHD history (%)	2.5	1.7 ± 0.1
Self-reported CVA history (%)	1.8	1.3 ± 0.1
Self-reported cancer history (%)	3.2	2.4 ± 0.1

Data are expressed as mean ± SEM, except for age with range. AUDIT, the Alcohol Use Disorders Identification Test; AUDIT scores were categorized into three groups: low-risk, 0 to 7 points; intermediate-risk, 8 to 15 points; and high-risk, ≥16 points; Hypertension, systolic blood pressure ≥ 140 mmHg, diastolic blood pressure ≥ 90 mmHg, or current anti-hypertensive medication; Diabetes, fasting plasma glucose ≥ 126 mg/dL, or current anti-diabetes medication or a previous diagnosis of diabetes by a doctor; HDL, high-density lipoprotein; CHD, coronary heart disease; CVA, cerebrovascular accident.

The weighted mean age of the participants was 45.0 years, and 49.8% of the participants were female. The mean resting HR was 69.8 beats per minute. The prevalence of hypertension was 25.9%, and 8.4% of participants were diabetic. The median duration of diabetes was 6 years (range 0–53).

### Weighted age- and sex-adjusted clinical characteristics according to resting HR

Table 2 presents weighted age- and sex-adjusted clinical characteristics according to four resting HR categories (HR ≤ 64, 65–69, 70–75, ≥ 76 bpm). Age had a tendency to decrease along with the increase in resting HR (P<0.001). The percentage of women increased across the four resting HR groups (P<0.001). Systolic BP (P<0.001), diastolic BP (P<0.001), the percentage taking anti-hypertensive medication (P = 0.006), and the prevalence of hypertension (P<0.001) showed significant trends across the HR groups. There was a significant positive relationship between resting HR and FPG level (P<0.001), the percentage of subjects taking anti-diabetes medication (P<0.001), IFG (P<0.001), and diabetes (P<0.001). Serum total cholesterol (P = 0.005), TG levels (P<0.001), and waist circumference (P<0.001) increased with the increase in resting HR. However, BMI was not associated with resting HR. High risk alcohol drinking (P = 0.002) was positively associated with resting HR. However, current smoking was not associated with resting HR.

**Table 2:** Weighted age- and sex-adjusted clinical characteristics according to resting heart rate.

Resting heart rate (bpm)	-64	65–69	70–75	76-	P
N (unweighted/weighted)	8,762/13,865,669	3,926/6,419,948	3,659/5,863,311	6,165/10,317,138	
Age (years)	47.0 (46.5–47.5)	44.9 (44.2–45.5)	44.7 (44.1–45.4)	42.7 (42.0–43.3)	<0.001
Women (%)	45.0 (44.0–46.0)	50.0 (48.0–52.0)	52.0 (50.0–54.0)	55.0 (54.0–57.0)	<0.001
Current smoking (%)	24.6 (23.5–25.7)	24.6 (22.9–26.2)	26.6 (24.8–28.5)	26.4 (25.0–27.7)	0.080
Alcohol drinking ≥ x4/week (%)	6.1 (5.4–6.7)	6.5 (5.5–7.4)	7.0 (6.0–8.1)	8.4 (7.5–9.3)	<0.001
High risk alcohol drinking (%)	10.5 (9.7–11.4)	11.5 (10.1–12.8)	12.8 (11.4–14.1)	12.9 (11.9–13.9)	0.002
Intermediate risk alcohol drinking (%)	21.0 (19.9–22.1)	20.8 (19.2–22.4)	20.8 (19.1–22.5)	19.9 (18.6–21.2)	0.650
Exercise ≥ x3/week (%)	19.8 (18.8–20.8)	19.6 (18.1–21.2)	18.5 (16.9–20.1)	17.0 (15.7–18.4)	0.004
College graduation (%)	32.6 (31.2–34.1)	33.1 (31.1–35.0)	31.1 (29.3–33.0)	31.4 (29.8–33.1)	0.306
Daily energy intake (kcal/day)	2102 (2074–2130)	2053 (2015–2090)	2086 (2044–2128)	2029 (1995–2062)	0.004
Body mass index (kg/m^2^)	23.7 (23.6–23.8)	23.7 (23.6–23.9)	23.8 (23.7–24.0)	23.6 (23.5–23.7)	0.204
Waist circumference (cm)	80.4 (80.1–80.7)	80.7 (80.3–81.1)	81.4 (81.0–81.8)	81.3 (81.0–81.7)	<0.001
Systolic BP (mmHg)	117.1 (116.7–117.5)	117.4 (116.8–118.0)	117.7 (117.1–118.3)	118.7 (118.2–119.2)	<0.001
Diastolic BP (mmHg)	75.1 (74.8–75.4)	76.4 (76.0–76.8)	76.7 (76.3–77.2)	77.3 (77.0–77.7)	<0.001
Anti-hypertensive Medication (%)	13.8 (13.1–14.6)	12.9 (11.8–13.9)	13.8 (12.6–15.0)	15.2 (14.3–16.0)	0.006
Hypertension (%)	24.0 (22.9–25.0)	24.5 (23.0–26.1)	26.3 (24.7–27.9)	29.2 (28.1–30.4)	<0.001
FPG (mg/dl)	94.8 (94.4–95.3)	96.6 (95.9–97.3)	97.5 (96.6–98.5)	100.6 (99.8–101.3)	<0.001
Anti-diabetes medication (%)	3.5 (3.1–4.0)	4.7 (4.1–5.4)	5.4 (4.6–6.2)	7.9 (7.2–8.7)	<0.001
IFG (%)	17.3 (16.3–18.3)	18.8 (17.2–20.3)	19.5 (18.0–20.9)	20.6 (19.4–21.8)	<0.001
Diabetes (%)	5.9 (5.3–6.4)	7.6 (6.7–8.5)	8.2 (7.1–9.2)	12.4 (11.5–13.3)	<0.001
Serum total cholesterol (mg/dl)	186.6 (185.6–187.6)	187.2 (185.9–188.6)	189.3 (187.8–190.8)	188.9 (187.7–190.1)	0.005
Serum triglyceride (mg/dl)	124.2 (121.7–126.7)	132.4 (128.3–136.4)	137.5 (132.1–142.9)	149.1 (144.9–153.3)	<0.001
Serum HDL-cholesterol (mg/dl)	52.7 (52.4–53.1)	52.5 (52.0–52.9)	52.5 (52.0–53.1)	52.0(51.7–52.4)	0.060
Serum WBC (/mm3)	5890 (5847–5932)	6066 (6009–6122)	6147 (6075–6219)	6460 (6400–6521)	<0.001
Serum hemoglobin (g/dl)	14.1 (14.1–14.1)	14.2 (14.2–14.3)	14.2 (14.2–14.3)	14.3 (14.2–14.3)	<0.001
Self-reported CHD history (%)	1.9 (1.6–2.3)	1.6 (1.2–2.0)	1.5 (1.1–2.0)	1.6 (1.3–1.9)	0.374
Self-reported CVA history (%)	1.2 (1.0–1.5)	1.1 (0.7–1.4)	1.2 (0.8–1.6)	1.6 (1.2–1.9)	0.170
Self-reported cancer history (%)	2.4 (2.0–2.7)	2.5 (1.9–3.0)	2.2 (1.7–2.7)	2.4 (2.0–2.8)	0.916

Data are expressed as mean with 95% CI. Hypertension, systolic blood pressure ≥ 140 mmHg, diastolic blood pressure ≥ 90 mmHg, or current anti-hypertensive medication; Diabetes, fasting plasma glucose ≥ 126 mg/dL, or current anti-diabetes medication or a previous diagnosis of diabetes by a doctor; FPG, fasting plasma glucose; IFG, impaired fasting glucose; HDL, high-density lipoprotein; WBC, white blood cell; CHD, coronary heart disease; CVA, cerebrovascular accident.

### The weighted and adjusted prevalence of diabetes according to resting HR

We assessed the weighted prevalence of diabetes according to resting HR (Table 3). After adjustment for age, sex, current smoking, high risk alcohol drinking, and daily energy intake (Model 1), the weighted prevalence rates of diabetes were 6.2% (5.6–6.8%), 7.3% (6.4–8.3%), 8.1% (7.0–9.2%), and 12.8% (11.8–13.9%) in subjects with HR ≤ 64, 65–69, 70–75, and ≥ 76 bpm, respectively (P<0.001). In Model 2, waist circumference, serum total cholesterol, serum TG, serum WBC, serum Hb, and the presence of hypertension, as well as age, sex, current smoking, high risk alcohol drinking, and daily energy intake were adjusted for the analysis. In Model 2, the weighted prevalence rates of diabetes were 6.8% (6.2–7.5%), 7.6% (6.7–8.5%), 8.0% (7.0–9.1%), and 11.8% (10.8–12.7%) in subjects with HR ≤ 64, 65–69, 70–75, ≥ 76 bpm, respectively (P<0.001). Adjustment for confounding factors in Model 3 was performed after excluding subjects taking anti-hypertensive medication in Model 2. In Model 3, the weighted prevalence of diabetes also increased across the HR groups (P<0.001). Results were similar in men and women.

**Table 3:** The adjusted weighted prevalence of diabetes according to resting heart rate.

Resting heart rate (bpm)	-64	65–69	70–75	76-	P
**Total population**					
N (unweighted/weighted)	8,762/13,865,669	3,926/6,419,948	3,659/5,863,311	6,165/10,317,138	
Model 1	6.2 (5.6–6.8)	7.3 (6.4–8.3)	8.1(7.0–9.2)	12.8 (11.8–13.9)	<0.001
Model 2	6.8 (6.2–7.5)	7.6 (6.7–8.5)	8.0 (7.0–9.1)	11.8 (10.8–12.7)	<0.001
Model 3					
N (unweighted/weighted)	6,916/9,874,242	3,220/4,826,812	2,980/4,292,370	5,059/7,556,806	
	4.1 (3.5–4.7)	5.1 (4.2–6.0)	5.1 (4.2–6.1)	8.0 (7.0–8.9)	<0.001
**Men**					
N (unweighted/weighted)	4,212/6,196,989	1,657/2,665,978	1,493/2,267,753	2,368/3,614,110	
Model 1	7.6 (6.7–8.5)	9.1 (7.5–10.7)	9.9 (8.1–11.7)	15.3 (13.5–17.1)	<0.001
Model 2	8.0 (7.1–9.0)	9.4 (7.8–11.0)	9.9 (8.1–11.6)	14.1 (12.3–15.9)	<0.001
Model 3					
N (unweighted/weighted)	3,372/5,270,881	1,353/2,337,848	1,239/1,979,603	1,922/3,109,692	
	5.4 (4.5–6.3)	7.2 (5.6–8.8)	6.9 (5.2–8.6)	10.5 (8.7–12.3)	<0.001
**Women**					
N (unweighted/weighted)	4,550/5,642,340	2,269/2,898,929	2,166/2,731,745	3,797/5,138,249	
Model 1	5.1 (4.3–5.9)	5.7 (4.5–6.9)	6.6 (5.4–7.8)	10.7 (9.5–11.9)	<0.001
Model 2	5.8 (5.0–6.6)	6.0 (4.9–7.2)	6.5 (5.4–7.7)	9.8 (8.7–10.8)	<0.001
Model 3					
N (unweighted/weighted)	3,544/4,603,361	1,867/2,488,964	1,741/2,312,767	3,317/4,447,114	
	3.1 (2.4–3.8)	3.2 (2.2–4.3)	3.6 (2.6–4.5)	5.7 (4.8–6.6)	<0.001

Model 1: adjusted for age, sex, current smoking, exercise ≥ x3/week (%), high risk alcohol drinking, and daily energy intake

Model 2: waist circumference, serum total cholesterol, serum triglyceride, serum white blood cell, serum hemoglobin, and the presence of hypertension and variables in model 1 adjusted

Model 3: model 2 after excluding subjects taking anti-hypertensive medication

### Weighted and adjusted resting HR according to FPG level

The unadjusted weighted resting HRs were 69.6, 69.4, 69.8, 70.1, and 72 bpm in the NFG 1, NFG 2, IFG 1, IFG 2, and diabetes groups, respectively (P<0.001) (Table 4, Model 1).

**Table 4:** The adjusted weighted resting heart rate (bpm) according to fasting plasma glucose level.

	NFG 1 (-89 mg/dl)	NFG2 (90–99 mg/dl)	IFG1 (100–110 mg/dl)	IFG2 (111–125 mg/dl)	diabetes	P
**Total population**						
N (unweighted/weighted)	7,763/13,437,127	7,980/13,106,956	3,314/5,080,328	1,187/1,789,964	2,268/3,051,691	
Model 1	69.6 (69.3–69.9)	69.4 (69.1–69.8)	69.8 (69.3–70.3)	70.1 (69.5–70.8)	72.0 (71.5–72.5)	<0.001
Model 2	68.7 (68.4–69.1)	69.5 (69.1–69.8)	70.6 (70.1–71.1)	71.4 (70.7–72.1)	73.7 (73.1–74.2)	<0.001
Model 3	68.7 (68.4–69.0)	69.4 (69.1–69.8)	70.4 (69.9–71.0)	71.5 (70.7–72.3)	73.6 (73.0–74.3)	<0.001
Model 4	68.9 (68.5–69.2)	69.6 (69.2–69.9)	70.3 (69.8–70.8)	70.9 (70.2–71.7)	72.9 (72.3–73.6)	<0.001
Model 5						
N (unweighted/weighted)	7.097/10,751,021	6,718/9,824,173	2,467/3,420,167	775/1,080,475	1,118/1,474,395	
	69.1 (68.8–69.5)	69.9 (69.5–70.3)	70.5 (69.9–71.1)	71.4 (70.5–72.3)	73.6 (72.7–74.5)	<0.001

Model 1: no adjustment

Model 2: adjusted for age and sex

Model 3: current smoking, exercise ≥ x3/week (%), high risk alcohol drinking, daily energy intake, and variables in model 2 adjusted

Model 4: waist circumference, serum total cholesterol, serum triglyceride, serum white blood cell, serum hemoglobin, and the presence of hypertension, and variables in model 3 adjusted

Model 5: model 4 after excluding subjects taking anti-hypertensive medication

NFG, normal fasting glucose; IFG, impaired fasting glucose.

We assessed the adjusted weighted resting HR according to the FPG level after adjusting for age, sex, smoking history, high risk alcohol drinking, daily energy intake, waist circumference, serum total cholesterol level, serum TG level, serum WBC, serum Hb, and the presence of hypertension (Table 4, Models 2–4). The adjusted weighted resting HR significantly increased across the FPG groups (P<0.001). After exclusion of subjects using anti-hypertensive medication in Model 4, we acquired the same results in Model 5.

### Effect of resting HR on the presence of diabetes

The ORs of resting HR for diabetes are shown in Table 5. Using resting HR ≤ 64 bpm as the control, resting HR ≥ 76 bpm was correlated with the presence of diabetes (adjusted OR 1.83, 95% CI 1.55–2.16, P<0.001). Each 10-bpm increment of HR increased the risk of the presence of diabetes by 35% (P<0.001).

**Table 5:** Logistic regression analyses for the presence of diabetes according to resting heart rate.

Resting heart rate	Crude odd ratio (95% CI)	*P*	Adjusted odd ratio (95% CI)	*P*
**Total population**				
-64 bpm	Reference		Reference	
65–69 bpm	1.09 (0.92–1.28)	0.322	1.13 (0.94–1.36)	0.197
70–75 bpm	1.15 (0.97–1.37)	0.099	1.16 (0.95–1.41)	0.154
75- bpm	1.66 (1.46–1.89)	<0.001	1.83 (1.55–2.16)	<0.001
Each 10 bpm increment	1.28 (1.21–1.34)	<0.001	1.35 (1.27–1.44)	<0.001
**Men**				
-64 bpm	Reference		Reference	
65–69 bpm	1.21 (0.98–1.50)	0.084	1.19 (0.92–1.54)	0.190
70–75 bpm	1.26 (1.01–1.59)	0.044	1.25 (0.95–1.63)	0.110
75- bpm	1.91 (1.60–2.29)	<0.001	1.92 (1.53–2.41)	<0.001
Each 10 bpm increment	1.36 (1.26–1.47)	<0.001	1.38 (1.25–1.52)	<0.001
**women**				
-64 bpm	Reference		Reference	
65–69 bpm	0.96 (0.76–1.22)	0.728	1.07 (0.81–1.40)	0.642
70–75 bpm	1.07 (0.84–1.35)	0.595	1.06 (0.81–1.39)	0.672
75- bpm	1.49 (1.23–1.81)	<0.001	1.77 (1.39–2.25)	<0.001
Each 10 bpm increment	1.22 (1.13–1.32)	<0.001	1.33 (1.21–1.47)	<0.001
**Younger adults**				
-64 bpm	Reference		Reference	
65–69 bpm	1.45 (0.89–2.35)	0.139	1.81 (1.03–3.10)	0.039
70–75 bpm	1.23 (0.74–2.04)	0.431	1.17 (0.65–2.12)	0.598
75- bpm	2.15 (1.45–3.20)	<0.001	2.54 (1.52–4.25)	<0.001
Each 10 bpm increment	1.42 (1.23–1.63)	<0.001	1.63 (1.34–1.98)	<0.001
**Middle-aged adults**				
-64 bpm	Reference		Reference	
65–69 bpm	1.25 (0.98–1.59)	0.074	1.02 (0.77–1.35)	0.902
70–75 bpm	1.59 (1.25–2.02)	<0.001	1.42 (1.07–1.88)	0.016
75- bpm	2.54 (2.08–3.09)	<0.001	2.14 (1.69–2.70)	<0.001
Each 10 bpm increment	1.51 (1.39–1.64)	<0.001	1.42 (1.29–1.56)	<0.001
**Older adults**				
-64 bpm	Reference		Reference	
65–69 bpm	1.11 (0.87–1.40)	0.405	1.11 (0.86–1.44)	0.414
70–75 bpm	0.97 (0.74–1.26)	0.810	0.92 (0.70–1.22)	0.577
75- bpm	1.76 (1.44–2.14)	<0.001	1.64 (1.32–2.05)	<0.001
Each 10 bpm increment	1.32 (1.22–1.43)	<0.001	1.30 (1.19–1.41)	<0.001
**Without anti-hypertensive medication**				
-64 bpm	Reference		Reference	
65–69 bpm	1.25 (1.01–1.56)	0.041	1.28 (1.00–1.64)	0.050
70–75 bpm	1.23 (0.98–1.54)	0.074	1.21 (0.93–1.57)	0.148
75- bpm	1.76 (1.47–2.10)	<0.001	1.95 (1.56–2.43)	<0.001
Each 10 bpm increment	1.31 (1.22–1.40)	<0.001	1.39 (1.27–1.53)	<0.001
**With anti-hypertensive medication**				
-64 bpm	Reference		Reference	
65–69 bpm	1.08 (0.84–1.39)	0.541	1.01 (0.77–1.31)	0.965
70–75 bpm	1.27 (0.99–1.64)	0.061	1.15 (0.87–1.52)	0.322
75- bpm	2.14 (1.74–2.64)	<0.001	1.83(1.44–2.33)	<0.001
Each 10 bpm increment	1.40 (1.29–1.53)	<0.001	1.34 (1.22–1.47)	<0.001

Adjusted for age, sex, current smoking, exercise ≥ x3/week (%), high risk alcohol drinking, daily energy intake, waist circumference, serum total cholesterol, serum triglyceride, serum white blood cell, serum hemoglobin, and the presence of hypertension

According to gender, resting HR ≥ 76 bpm was also associated with the presence of diabetes in both men (adjusted OR 1.92, 95% CI 1.53–2.41, P<0.001) and women (adjusted OR 1.77, 95% CI 1.39–2.25, P<0.001), compared to resting HR ≤ 64 bpm.

Concerning age, in middle-aged adults (45–64 years of age), resting HR 70–75 bpm (adjusted OR 1.42, 95% CI 1.07–1.88, P = 0.016) was correlated with the presence of diabetes, as was resting HR ≥ 76 (adjusted OR 2.14, 95% CI 1.69–2.70, P<0.001), using resting HR ≤ 64 bpm as the control. In younger (10–44 years of age) or older (≥ 65 years of age) adults, diabetes was associated only with resting HR ≥ 76 bpm, compared to resting HR ≤ 64 bpm.

The association of high resting HR with the presence of diabetes was not influenced by BP medication status.

## Discussion

Using data from KNHANES 2010–2013, we demonstrated that higher HR was associated with diabetes in a representative sample of Korean adults. The positive associations between HR and diabetes in this study were independent of several factors influencing HR, including age, sex, current smoking, high risk alcohol drinking, daily energy intake, waist circumference, and the presence of hypertension.

Previously, a few studies have evaluated the relationship between HR and diabetes in non-Western populations. Zhang *et al*. demonstrated that a high resting HR was independently associated with a moderately increased risk of type 2 diabetes in healthy Chinese women.[10] Li *et al*. demonstrated that a fast resting HR is associated with an increased risk of undiagnosed diabetes in a Chinese male and female adult population [22]. A prospective study including healthy Japanese people suggested that HR > 80 bpm had a significant predictive value for the development of diabetes 20 years later [11].

A recent meta-analysis showed that the relative risk of diabetes for each 10 bpm increment in resting HR was 1.2 (95% CI: 1.07–1.34, N = 9), although there was substantial heterogeneity [9]. Similarly, we found that each 10-bpm increment of HR increased the risk of the presence of diabetes by 36% (P<0.001) in this study. Furthermore, those who have IFG prior to diabetes also have a significantly higher resting HR compared to subjects with NFG. Wang *et al*. reported that faster resting HR is associated with higher risk of developing IFG, suggesting that HR could be used to identify individuals with a higher future risk of diabetes [23]. However, the weighted prevalence rates of IFG according to resting HR did not show significant differences after adjustment for potential confounding factors in our study.

Yang *et al*. also examined the association of resting heart rate with hypertension, diabetes, and metabolic syndrome, using 2010–2012 KNHANES, similar to our study [17]. However, we focused the relationship between resting heart rate and the presence of diabetes in more detail. While Yang *et al*. showed the odds ratio for the prevalence of diabetes according to gender and resting heart rate, our study demonstrated the odds ratio for the presence of diabetes according to the age group and anti-hypertensive medication status, as well as gender, and resting heart rate. Because elevated resting heart rate could be a consequence, and equally, a marker reflecting risk factors of developing diabetes, we tried to adjust for potential confounding factor as many as possible. Compared with previous study performed by Yang *et al*. adjusting for known confounding factors such as age, BMI, physical activity, smoking and alcohol consumption, we additionally adjusted for the daily energy intake, serum total cholesterol, serum triglyceride, serum WBC, serum hemoglobin and waist circumference instead of BMI.

In this study, we confirmed and adjusted for various factors including physiologic and pathologic conditions that influence resting HR.

Most studies have shown that resting HR progressively declines with aging [2]. Our study also showed that the average age decreased with increases in the resting HR. Recent data suggest that such a change is largely explained by decreases in both intrinsic HR and chronotropic beta-adrenergic responsiveness [24].

The proportion of women also tended to increase with resting HR in this study, which is consistent with a previous finding that HR was higher in women than in men [2,25]. Women have higher parasympathetic and lower sympathetic drive of HR than men and a higher set point for HR around which the autonomically modulated frequency oscillates [25].

Several studies reported an association between HR and body weight [11,26,27]. Long-sustained increased sympathetic tone might induce a down-regulation of adrenergic metabolic and energy expenditure responsiveness, predisposing an individual to obesity [26,28]. However, we could not find a significant association between BMI and HR. We think that this is probably due to the relatively low prevalence of obesity (32.2%; 95% CI, 31.4–33.0) for BMI ≥25 kg/m^2^) in our population compared to Western populations.

Recently, emerging evidence has shown that resting HR could predict metabolic syndrome as well as diabetes [29–31]. Systolic and/or diastolic BP, waist circumference, and serum TG level, which are components of metabolic syndrome, also significantly increased along with the increase in resting HR in this study.

Finally, smoking and alcohol habits are also important lifestyle determinants of resting HR. In a nationwide Belgian survey, cigarette smoking was the second strongest determinant of resting HR after BP in men [32]. This association is mediated by the stimulation of peripheral adrenergic receptors as a result of increased plasma catecholamines [33]. In this study, the percentage of current smoking was higher in subjects with resting HR ≥ 76 bpm than those who had a resting HR < 64 bpm (p = 0.043). However, there was no significant trend across the four resting HR categories. Heavy alcohol consumption is also known to increase HR, which is consistent with our study, although the effect of moderate daily alcohol intake on HR is known to be negligible [2].

Although we did not elucidate the mechanism of the association between elevated HR and diabetes in this study, we consider that diabetic cardiac autonomic neuropathy has an important role in this association.

Diabetes can lead to dysfunction in the autonomic nervous system, including cardiac autonomic neuropathy, which is caused by impairment of the autonomic nerve fibers regulating heart rate, cardiac output, myocardial contractility and cardiac electrophysiology[34]. Because neuropathy first affects the longest nerve fiber, the first manifestation of diabetic cardiac autonomic neuropathy tends to be related with vagus nerve damage, which is responsible for nearly 75% of parasympathetic activity [35]. This damage causes resting tachycardia as the sympathetic activity becomes dominant [36].

Inversely, sympathetic over-activation has been shown to contribute to insulin resistance via both hemodynamic and cellular effects [37]. Sympathetic activation causes vasoconstriction and decreases skeletal muscle blood flow, resulting in the impairment of glucose uptake into the skeletal muscle [38]. Furthermore, sympathetic over-activation might influence decreased insulin secretion as well as insulin resistance. The pancreas is extensively innervated by parasympathetic nerve fibers that stimulate insulin secretion from β-cells in response to blood glucose levels, and sympathetic over-activation can inhibit insulin secretion [39].

Second, because the autonomic nervous system plays a role in the inflammatory response, higher HR is significantly associated with increased inflammatory responses [40]. It is recognized that subclinical chronic inflammation is involved in the pathogenesis of the development of diabetes [41]. Vinik described activation of inflammatory cytokines like IL-6 and TNFα in newly diagnosed type 2 diabetes and that the inflammatory change correlates with abnormalities in sympathovagal balance [42]. Although we could not analyze inflammatory cytokines or C-reactive protein, we found that serum WBC increased significantly across the HR groups, supporting this mechanism.

The major strength of our study is that we demonstrated an association between higher HR and diabetes in a large nationally representative sample of Korean adults. Additionally, this is the first nationwide study to show that the adjusted weighted resting HR significantly increased across the FPG groups prior to diabetes.

Nevertheless, this study had some limitations. First, because this was not a prospective observational study, we could not evaluate the actual incidence of diabetes. Thus, we could not draw an inference regarding causality due to the cross-sectional design of the study. Second, residual or hidden confounding variables cannot be excluded, as with other cross-sectional studies. Third, we did not have data about specific use of beta blockers, which reduce HR and increase the risk of developing diabetes. However, analyses after exclusion of subjects taking anti-hypertensive medication showed similar results, suggesting that concomitant use of anti-hypertensive medication, including beta blockers, is not likely to have had a significant effect on our results. Fourth, we could not evaluate congestive heart failure or cardiac arrhythmia that would affect resting heart rate, because KNHANES do not include data about these disorders. Finally, KNHANES did not include random glucose level, so we could not use random glucose level for diagnosing diabetes. Furthermore, HbA1c was measured only for the subject who had the medical history of diabetes in KNHANES 2010, we could not use HbA1c as a diagnostic tool for diabetes in this study, using data from the 2010–2013 KNHANES.

In conclusion, higher HR was associated with diabetes in a representative sample of Korean adults. We suggest that individuals with a higher resting HR are at risk of diabetes and that HR might provide an easy and simple surrogate marker for the risk of diabetes.
